# Immune-Related Disorders and Extrahepatic Diseases in Chronic HCV Infection

**DOI:** 10.1155/2012/509309

**Published:** 2012-10-11

**Authors:** Domenico Sansonno

**Affiliations:** Liver Unit, Department of Biomedical Sciences and Human Oncology, Medical School, University of Bary, Piazza G. Cesare, 11 70124 Bari, Italy

Hepatitis C virus (HCV) represents one of the most important causes of chronic active liver disease worldwide, potentially resulting in cirrhosis, and hepatocellular carcinoma. 

HCV is mainly characterized by two major immunologic fingerprints, namely, escape of immune response in more than 80% of infected patients and production of autoantibodies in almost half of them. 

Within the infected host, HCV exists as a *quasispecies *or a flock of related viral sequences. HCV can evolve rapidly as its replicative cycle yields 10^12^ HCV particles per day in the liver, whereas in the serum the newly produced viral particles have an estimated halflife of about 3 hours. This high rate of virus production coupled with the lack of proofreading capacity of the HCV's NS5B RNA-dependent RNA polymerase lead to its rapid evolution and escape from immune recognition in each host. In addition, HCV profoundly deranges the functions of immune system. It deregulates both innate and adaptive antiviral response. It has been shown that HCV-encoded proteins subvert type I interferon (IFN) receptor signal transduction and the function of downstream IFN effector pathway. The virus decreases the ability of NK cells to promote dendritic cell maturation modifying antigen presentation and production of soluble mediators including IL-10 and IL-12. Resolution of HCV infection is mainly dependent on adaptive immunity and is associated with a robust and sustained specific T-cell response targeting multiple epitopes and intrahepatic IFN-*γ* production. Defective CD4^+^ T cells in both acute and chronic HCV infection lead to CD8^+^ T-cell exhaustion. 

While HCV evolves under immunological pressure, the cellular immune response remains focused on viral sequences encountered early in the course of the infection. Indeed, humoral immune response seems more flexible in that the continued emergence of new antibody specificities sharply contrasts with the static T-cell response. Indubitable, interaction of HCV with B cells may promote favourable conditions for lymphocyte proliferation. Viral replicative intermediary was found in the B cell from patients with mixed cryoglobulinemia (MC), whereas no traces of HCV productive particles were demonstrable in HCV-infected individuals without cryoglobulin production, supporting the notion that HCV is not a genuine lymphotropic virus, but its entry and replication are largely dependent on host selective interactions. HCV binding to BCR triggers both the initiation of signaling cascade and internalization of the BCR and bound antigen into the cell. These interactions result in *in vivo *polyclonal activation and expansions of CD19^+^ CD5^+^ cells.

 Extrahepatic disease manifestations, which include autoimmune phenomena and frank autoimmune and/or rheumatic diseases, may complicate the clinical features of chronic HCV infection and sometimes dominate its course in almost half of chronic HCV carriers. Possibly, progression to frank B-cell lymphoid malignancy may also be superposed as additional stochastic oncogenic event. However, there are many dark areas in the comprehension of several aspects of their pathogenetic mechanisms. The nature of the process during which B cells expand with preferential involvement of rheumatoid factor- (RF-) producing B cells is undefined. Whether B-cell clonal expansion of a particular specificity occurs as a result of distinct selection is not clear. The process of B-cell clonal expansion occurring in an environment favourable to the immortalization of one specific clone must be clarified. Predisposing factors for transforming events must be identified. 

Against this background, it was felt that times were ripe to produce a state-of-the-art survey of the multifaceted pictures of HCV-related immune disorders. This special issue is therefore devoted to expand our knowledge on the features and mechanisms underlying the relationship between B-cell immune response and extrahepatic manifestations of chronic HCV infection. It comprises 16 review articles and 1 clinical study.

 The paper by T. Himoto and T. Masaki is a general overview on HCV-related extrahepatic manifestations including cryoglobulinemia, Sjögren's syndrome, autoimmune thyroid disease, lichen planus, CREST syndrome, IFN-induced autoimmunity, and autoimmune cytopenia. Few data are available on this topic in children. The paper by G. Indolfi et al. deals with the clinical significance of non-organ specific autoantibodies in the course of paediatric chronic hepatitis C. Subclinical hypothyroidism and membranoproliferative glomerulonephritis have been described. 

Dysregulation of the cytokine/chemokine network, involving proinflammatory and Th1 chemokines, is addressed by P. Fallahi et al. Both the humoral and viral counterparts at the basis of cryoglobulins production in HCV-induced type II MC, with particular attention to the most frequently involved single IgV subfamilies have been analyzed by G. Sautto et al. Neurological complications that occur in a large number of patients and range from peripheral neuropathy to cognitive impairment are discussed by S. Monaco et al. Autoimmune thrombocytopenia is a frequent complication of HCV chronic infection. D. Dimitroulis et al. estimated the epidemiological characteristics of the disease and discussed the potential treatment strategies. Extrahepatic manifestations of HCV infection include a multitude of disease processes affecting the small vessels, skin, kidneys, salivary gland, eyes, thyroid, and immunologic system. The majority of these conditions are thought to be immune-mediated. H. M. Ko et al. highlighted the histomorphologic features of these pathologic conditions. G. Lauletta et al. described epidemiological, clinical, and pathogenetic mechanisms underlying the cryoglobulinemic vasculitis. In MC, consumption of complement component 4 may be due to activation of complement cascade. Potential activators include monoclonal IgM-RF, IgG antibodies, and the complexing of the two in the cold, perhaps modulated by the rheology and stoichiometry of cryocomplexes in specific microcirculations. P. D. Gorevic elucidated the role of the RF and the complement in the vessel damage. 

The association between HCV infection and B-cell non-Hodgkin's lymphomas has led to search for molecular signatures capable of predicting patients' characteristics. V. De Re et al. underscored that HCV-related lymphomas are subject to specific deregulation induced by the virus. Epidemiology and mechanisms of HCV-induced lymphoproliferation have been elucidated by F. Forghieri et al. Different biological mechanisms, namely, chronic antigen stimulation, high-affinity interaction between HCV-E2 protein and its cellular receptors, direct HCV infection of B cells, and “hit and run” transforming events, may cooperate in a multifactorial model of HCV-associated lymphomagenesis. A comprehensive review of molecular mechanisms involved in HCV-related lymphomagenesis has been resumed by A. L. Zignego et al. It is concluded that HCV lymphomagenesis is a complex, multistep, multifactorial process. L. Arcaini et al. illustrated the relationship between HCV infection and different subtypes of indolent B-cell lymphomas. They summarized the data from the therapeutic studies reporting the use of antiviral treatment as hematological therapy. L. Chiche et al. provided an updated overview on the place of immunotherapy, especially biologics, in the management of HCV-induced cryoglobulinaemic vasculitis. Rituximab has been used to treat oncohaematological diseases, B-cell-related autoimmune diseases, rheumatoid arthritis, and, more recently, HCV-associated mixed cryoglobulinaemic vasculitis. E. Sagnelli et al. discussed rituximab-based treatment and conclude that this drug is capable of enhancing HCV replication with potential liver failure. Y. Kishida et al. explored the hypothesis that an induction approach with nIFN-beta followed by PEG-IFN-alpha plus ribavirin would increase the initial virologic response in chronic hepatitis C. They concluded that this combined therapeutic scheme results in high rate of sustained virologic response and improvement of innate and adaptive immunity. F. Bellanti et al. accomplished an overview about the biological activity and clinical applications of interferon lambda 3, summarizing the available data on its impact on HCV infection. The potential usefulness of this type of interferon in the treatment of HCV infection has been also discussed.

*Domenico Sansonno*

